# An efficient method for zoospore production, infection and real-time quantification of *Phytophthora cajani* causing Phytophthora blight disease in pigeonpea under elevated atmospheric CO_2_

**DOI:** 10.1186/s12870-015-0470-0

**Published:** 2015-03-25

**Authors:** Mamta Sharma, Raju Ghosh, Avijit Tarafdar, Rameshwar Telangre

**Affiliations:** Legumes Pathology, International Crop Research Institute for the Semi-Arid Tropics, Patancheru, 502324 Telangana India

**Keywords:** Phytophthora stem blight, Inoculation technique, Elevated CO_2_, qPCR

## Abstract

**Background:**

Phytophthora blight caused by *Phytophthora cajani* is an emerging disease of pigeonpea (*Cajanus cajan* L.) affecting the crop irrespective of cropping system, cultivar grown and soil types. Current detection and identification methods for *Phytophthora* species rely primarily on cultural and morphological characteristics, the assessment of which is time-consuming and not always suitable. Sensitive and reliable methods for isolation, identification, zoospore production and estimating infection severity are therefore desirable in case of Phytophthora blight of pigeonpea.

**Results:**

In this study, protocols for isolation and identification of Phytophthora blight of pigeonpea were standardized. Also the method for zoospore production and *in planta* infection of *P. cajani* was developed. Quantification of fungal colonization by *P. cajani* using real-time PCR was further standardized. *Phytophthora* species infecting pigeonpea was identified based on mycological characters such as growth pattern, mycelium structure and sporangial morphology of the isolates and confirmed through molecular characterization (sequence deposited in GenBank). For Phytophthora disease development, zoospore suspension of 1 × 10^5^ zoospores per ml was found optimum. *Phytophthora* specific real-time PCR assay was developed using specific primers based on internal transcribed spacer (ITS) 1 and 2. Use of real-time PCR allowed the quantitative estimation of fungal biomass in plant tissues. Detection sensitivities were within the range of 0.001 pg fungal DNA. A study to see the effect of elevated CO_2_ on Phytophthora blight incidence was also conducted which indicated no significant difference in disease incidence, but incubation period delayed under elevated CO_2_ as compared to ambient level.

**Conclusion:**

The zoospore infection method for Phytophthora blight of pigeonpea will facilitate the small and large scale inoculation experiments and thus devise a platform for rapid and reliable screening against Phytophthora blight disease of pigeonpea. qPCR allowed a reliable detection and quantification of *P. cajani* in samples with low pathogen densities. This can be useful in early warning systems prior to potential devastating outbreak of the disease.

## Background

Pigeonpea (*Cajanus cajan* (L.) Millsp.) is one of the important legume crops of rainfed agriculture in the semi-arid tropics. The Indian subcontinent, Eastern Africa and Central America, three main pigeonpea producing regions of the world cultivates pigeonpea either as a sole crop or intermixed. As pigeonpea contains high level of protein and some essential free amino acids like methionine, lycine and tryptophan, the importance of the pigeonpea to world vegetarian population is very significant. In India, pigeonpea is second most important legume after chickpea and alone contributes 72.5% of world cultivated area with 62.5% of world production [1]. The accelerated susceptibility of pigeonpea to diseases in Indian subcontinent is one of the main roots of its deteriorating productivity.

Phytophthora blight (PB), caused by *Phytophthora cajani* is an emerging disease in pigeonpea [2,3]. Information on total economic losses in India caused by PB is not available, but the disease is of rising importance since last a few years and has the potential to cause 100% yield losses in field conditions under favourable environment. Occurrence and widespread distribution of PB has been reported in areas especially when excessive rains fall within a short span of time and hot and humid weather persists during the crop season [2]. There is a lack of comprehensive knowledge on available resistant genotypes to *P. cajani* [3].

Identification of *Phytophthora* species by conventional diagnostic tests abased on morphology, and growth on selective media is time consuming, laborious and takes considerable skill. In addition, taxonomic expertise is required for correct identification within the closely related species. Again the prospective for quantification of biomass of pathogen is limited. In this regard, the aim of the study was also to develop a rapid, highly specific and very sensitive method for the potential quantification of *P. cajani.* The polymerase chain reaction (PCR) has long been used to detect the pathogens and is a highly sensitive and relatively fast method that allows detecting specific target DNA molecules in a complex mixture, offering an alternative to microbiological conventional procedures in fungal diagnostic. One of the most important factors in the development of such molecular methods is the reliability of the primer set and the targeted DNA sequence of interest organism [4]. Nuclear rDNA including the small and large subunits, 5.8S and the internal transcribed spacer (ITS) region, proved to be an ideal target for specific PCR primers, as each sequences is variable at the family, genus or species level [5]. The ITS region has been shown to be largely conserved within *Phytophthora* spp. but differ across species [6,7]. Most importantly, sequence information is available in this region for nearly all known species of *Phytophthora* [6]. Consequently, we designed *P. cajani* specific primers within the ITS region. The current technique has the further advantage of being able to be performed as real-time PCR, visualized using an intercalating dye such as SYBR green. Real-time PCR allows products to be distinguished based not only size but also on sequence, because melt temperatures will differ for same size but distinct products [8,9].

In order to establish a successful method for *in vitro* infection of *P. cajani*, a standardised protocol is needed to culture at its pathogenic state and to isolate the zoospores. However, knowledge about the conditions which govern infection by zoospores of *P. cajani* is yet unknown. Several workers have described inoculation techniques using mycelium [10,11], sporangium [12] and zoospores [13,14] for obtaining infection with *Phytophthora* spp.; however *P. cajani* being a putatively novel species, very limited information about laboratory protocols are available. Further, Amin et al. [15] recognized that *P. cajani* resembled *P. drechsleri* but noted that *P. cajani* produced larger sporangia and undifferentiated sporangiophores. They also considered that the homothallic nature of *P. cajani* differentiated it from *P. drechsleri*, which is considered to be heterothallic according to Savage et al. [16]. However, Kannaiyan et al. [17] expressed the opinion that PB caused by *P. drechsleri* var. *cajani* and *P. cajani* are probably the same. Detailed descriptions for both these pathogens have been provided in Phytophthora Diseases Worldwide by Erwin and Ribeiro [18]. In current study, attempts have been made to establish an *in planta* infection system of *P. cajani* in pigeonpea.

Host and pathogens are influenced by the interactive effects of multiple climatic factors. If any one of the factors of disease triangle (host, pathogen and environment) is altered, changes in the progression of a disease epidemic can occur [19]. High humidity, rising temperature, elevated CO_2_ and depletion of O_3_, all is events of imperative deviations in atmospheric components. Changes in these important climatic factors play a direct or/and indirect role in changes of pathosystem and in disease expression [20]. Anthropogenic emissions are drastically increasing the concentration of atmospheric CO_2_, as in 1750 atmospheric CO_2_ was 280 ppm has increased to 400 ppm in 2013 and the projected concentration to be1250 ppm by 2095 [21]. Elevated CO_2_ directly alters growth, development, metabolism and plant physiology which, in turn, has an impact on pathogen invasion and disease progress [22]. Elevated CO_2_ can also modify plant–pathogen interactions primarily through changes in host plants [23].

Till now it is not clear in the literature, whether the disease severity is enhanced or diminished by a higher CO_2_. The study of Thompson and Drake [24] showed that elevated CO_2_ reduced powdery mildew (*Erysiphe graminis*) on wheat and the severity of rust (*Puccinia sparganioides* Ellis & Barth) on C3 sedge (*Scirpus olneyi*) Grey. On the other side, higher temperature and increased CO_2_ concentration are also posing higher threat by increasing fungal disease perception namely late blight of potato, rice blast and sheath blight. In this context, there is a need to study effect of elevated CO_2_ on Phytophthora blight of pigeonpea, as in the last decade the disease dynamics of pigeonpea has changed drastically in India.

The overall objective of the study was to (i) standardize protocol for isolation and infection of *P. cajani,* (ii) assess the effect of elevated atmospheric concentration of CO_2_ on PB development and (iii) quantify *P. cajani* during *in planta* infection.

## Results

### Isolation of *Phytophthora*

Pigeonpea plants with typical symptoms of Phytophthora blight were sampled from the infected fields of multiple locations of two different states of India during 2012-2013 and 2013–2014. Blight symptoms included brown to dark brown lesions distinctly different from healthy green portions on main stem, branches and petioles. These lesions sometimes elongate and cause girdle and cracks on the stems. Varying levels of PB incidence was recorded in surveyed locations (5.25-69.9%) irrespective of years. There was no effect of soil type on PB incidence (Table 1). A total of 14 isolates of *Phytophthora* (nine from 2012–2013 and five from 2013–2014), one isolate from Varanasi-Uttar Pradesh, one isolate from each district of Mahbubnagar and Adilabad of Telangana State and rest 11 isolates from different experimental fields (BP5, BP09, BP14, BP15, DHF4, RL17 and RM1) of ICRISAT were obtained (Table 1). The pure culture of the isolates was maintained on 20% tomato extract agar media at 15 ± 1°C in dark condition*.*

**Table 1:** **Passport information of** ***Phytophthora cajani*** **isolates used in the study**

S. no*	**Isolates code**	**Sample site and year of collection**	**Location**	**Soil type**	**PB incidence (%)**	**Length of ITS sequence (nt) submitted**	**Accession number**
1	ICPC 1	RL17-2013	Patancheru, Telangana	Red	43.75	504	KJ010534
2	ICPC 2	BP09-2013	Patancheru, Telangana	Black	10.20	534	KJ010535
3	ICPC 3	RM1-2013	Patancheru, Telangana	Red	5.25	728	KJ010536
4	ICPC 4	BP5-2013	Patancheru, Telangana	Black	7.59	698	KJ010537
5	ICPC 5	BP15-2013	Patancheru, Telangana	Black	12.50	518	KJ010538
6	ICPC 6	Mahbubnagar-2013	Telangana	Black	24.40	772	KJ622200
7	ICPC 7	Adilabad-2013	Telangana	Black	69.90	570	KJ622201
8	ICPC 8	DHF4-2013	Patancheru, Telangana	Red	58.74	698	KJ622202
9	ICPC 9	Varanasi-2013	Uttar Pradesh	Sandy loam	48.40	467	KJ622203
10	ICPC 10	BP14-2014	Patancheru, Telangana	Black	18.31	686	KJ622204
11	ICPC 11	BP14-2014	Patancheru, Telangana	Black	13.04	506	KJ622205
12	ICPC 12	BP14-2014	Patancheru, Telangana	Black	25.00	687	KJ622206
13	ICPC 13	BP14-2014	Patancheru, Telangana	Black	5.77	749	KJ622207
14	ICPC 14	BP14-2014	Patancheru, Telangana	Black	14.29	822	KJ622208

*Serial number.

### Morphological identification

**Figure 1:**
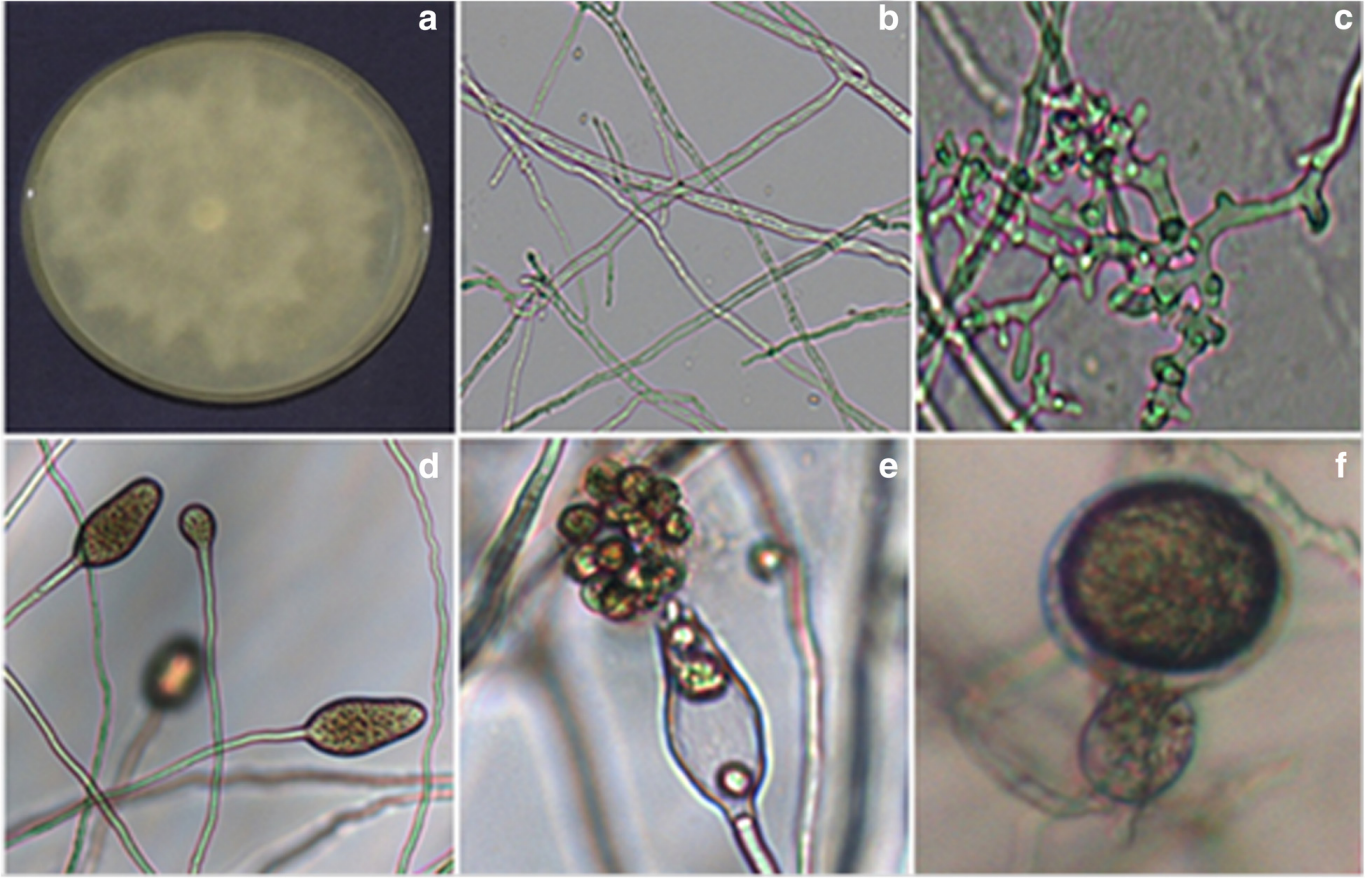
**Morphology of** ***Phytophthora cajani*** **isolate ICPC 1: (a)** growth in Petriplate; **(b)** coenocytic mycelium; **(c)** hyphal swellings; **(d)** non-papillate sporangia; **(e)** zoospores; **(f)** oospore.

The growth pattern, mycelium structure and sporangial morphology of the individual isolates of *Phytophthora* were examined under microscope for identification of the species. Pathogen formed distinct colonies in rosaceous, stellate or cottony pattern irrespective of isolates. The mycelium of the isolates was coenocytic and branched. Some isolates formed hyphal swellings on V8 agar. Karyotyping of the isolates showed single or pair of nuclei within the hyphae. Sporangium produced by different isolates varied in structure from broadly ovoid, obpyriform to elongate and non papilate. Based on morphological characters, the pathogen was identified as *Phytophthora cajani* (Figure 1a-f). Amphigynous anthredia were also observed and resembled to *Phytophthora cajani* as given in the manual of Phytophthora: Identifying species by morphology and DNA fingerprints [25].

### Molecular characterization

**Figure 2:**
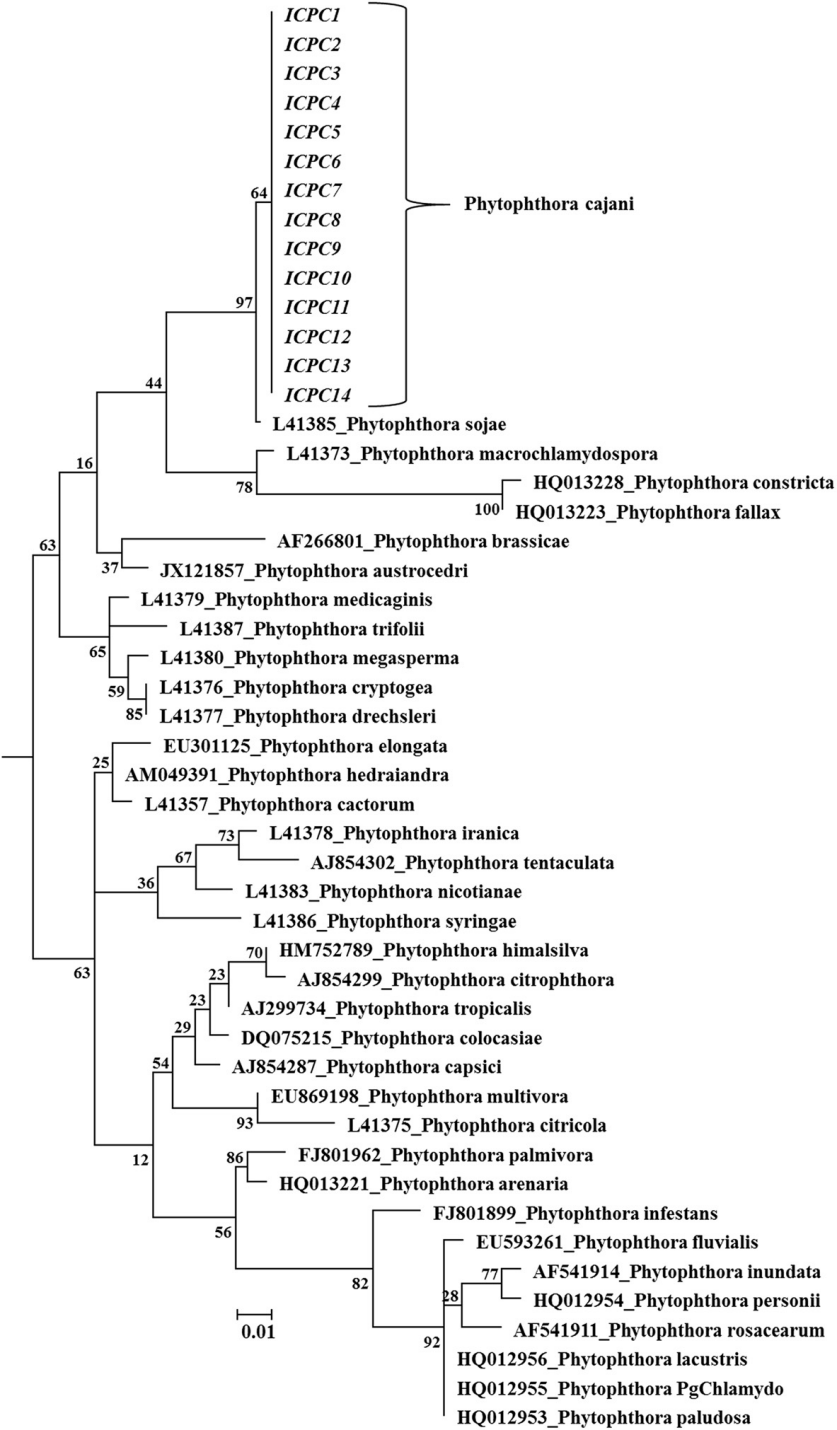
**The maximum likelihood phylogenetic tree showing the relationship among the** ***P. cajani*** **isolates of this study and other** ***Phytopthora*** **spp. based on ITS sequences of 5.8S rDNA.** Scale bar represent the genetic distance, proportional to the number of nucleotide differences between branch nodes. The significance of the nodes was estimated with 1000 bootstrap repetitions. The isolates of the current study is marked by Italics font.

To confirm the *P. cajani* at molecular level, the ITS sequence of 5.8S rDNA of the 14 isolates was amplified and sequenced. The sequences were purified and were used in a BLASTn against the Phytophthora database (http://www.phytophthoradb.org/). The BLAST result confirmed the identity of the isolates as *P. cajani*. The sequences were deposited under the GenBank (Table 1). The pair-wise nucleotide sequence identity matrix showed 99-100% sequence identity among the isolates. The phylogenetic relationship among the isolates and as well as with other *Phytophthora* spp. was made based on nucleotide sequences of ITS region. In phylogenetic tree, it was found that all the isolates of *P. cajani* were grouped together in the same clade with the nearest relative *P. sojae* isolate. There was no phylogenetic discordance within the *P. cajani* isolates (Figure 2).

### Zoospores production and *in planta* infection

Pathogenicity of all the 14 isolates was tested individually on the susceptible cultivar of pigeonpea ICP 7119. The pathogenicity of the isolates was tested on 50 seedlings. The complete mortality of seedlings was recorded two days after the inoculation. Lesions were seen on the stem and whole seedlings collapsed within 6–7 days after inoculation. All the 14 isolates were found to be virtually equal pathogenic to host ICP 7119 (data not shown). The *P. cajani* isolate ICPC 1 was taken to develop a method for zoospores production and *in planta* infection as it had shown consistent susceptible reaction. We developed the reproducible method for zoospore production from *P. cajani* and the method of inoculation for rapid screening for resistance to PB in pigeonpea genotype. The zoospores produced by *P. cajani* isolate ICPC 1 were measured under haemocytometer and inoculum concentration of 1.5 × 10^5^ zoospores per ml was found to be optimum for PB development. The symptoms started after 2 days of inoculation and 100% seedling mortality was recorded at 3–4 days post inoculation. Un-inoculated plants did not show any disease symptom throughout the tests.

### PB incidence under elevated CO_2_

**Figure 3:**
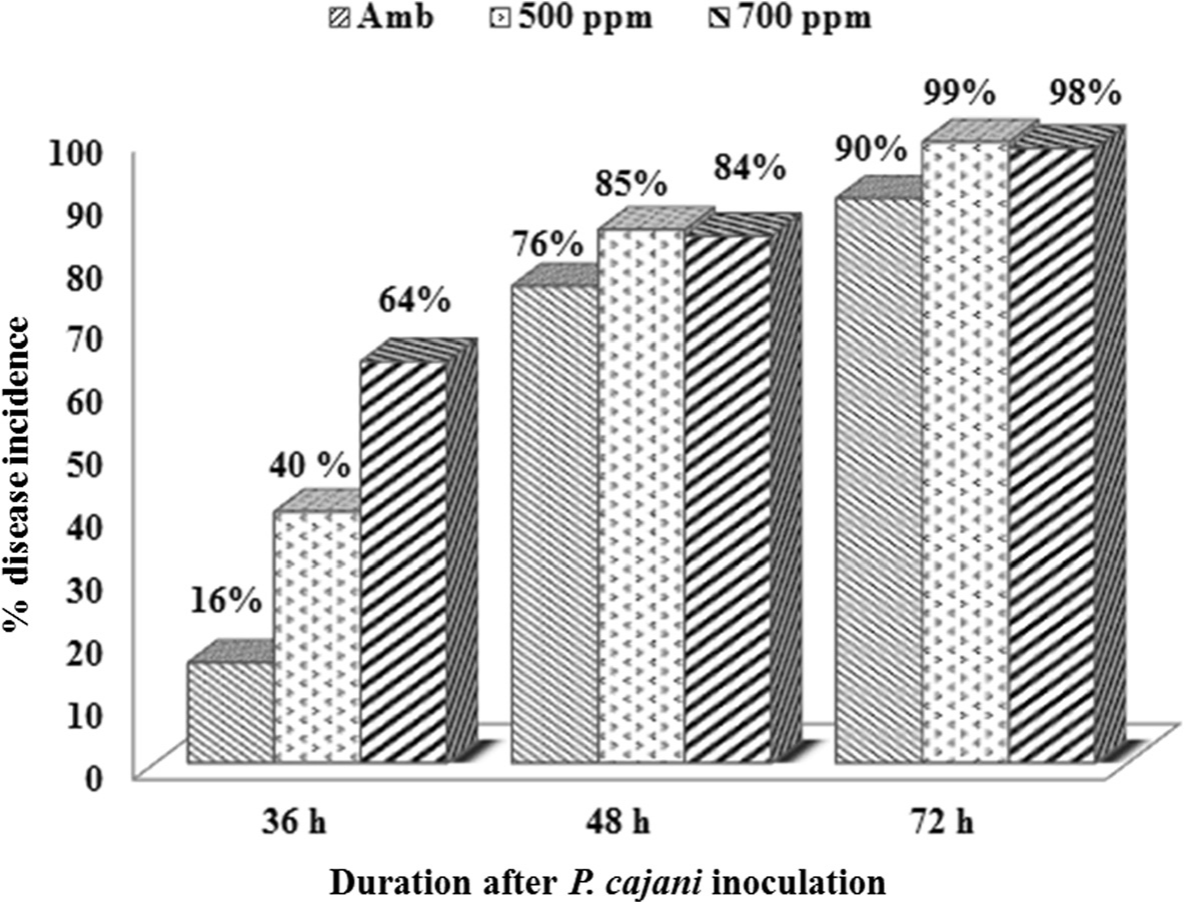
**Comparison on progression of PB incidence under ambient condition and elevated CO**_**2**_ **.**

Incubation period, measured as the time in days between inoculation and disease symptoms was significantly delayed under elevated CO_2_ as compared to ambient (*p* < 0.001). PB symptoms appeared after 30 hours of inoculation under ambient condition, after 36 hours under elevated CO_2_ levels of 550 ppm and 40 hours under 700 ppm but, it was observed that disease progressed faster under elevated CO_2_ as compared to ambient. No significant effect of ambient and elevated CO_2_ concentrations on PB disease incidence was observed after a certain period of inoculation (48 hours and 72 hours after inoculation) (Figure 3).

### Development of qPCR for the quantification of *Phytophthora cajani*

#### Specificity of the primers

The specificity of the qPCR primers designed from ITS sequences of ribosomal DNA was assessed using DNA from targeted *Phytophthora* and non-targeted other fungal species such as *Fusarium udum, Macrophomina* sp, *Alternaria alternata*. The assessment showed that the specific primers, qPCR_F2 and qPCR_R2 used in this study only amplified 146 bp PCR products from targeted species, *P. cajani* (Table 2). The PCR product was sequenced and sequence obtained from the amplicon was aligned with targeted sequence. Specificity of the primers was confirmed by its 100% similarity with *P. cajani*. No signal was generated from any non-targeted fungal pathogen indicating excellent specificity and sensitivity of the primers.

**Table 2:** **Details of the PCR primers used in this study**

**S. No.**	**Primers name**	**Primer sequence (5' → 3')**	**PCR T**_**m**_ **(°C)**	**Product size (bp)**	**Used for**	**Respond/Remarks**
Primers pair 1	ITS 1	TCCGTAGGTGAACCTGCGG	55	~800	Identification of *P. cajani* by amplification of ITS sequence	Amplified and PCR products sequenced
	ITS 4	TCCTCCGCTTATTGATATGC				
Primers pair 2	qPCR_F1	CTTTCAGCAGTGGATGTCTAGG	62	127	qPCR quantification of *P. cajani*	Low reproducible results in qPCR
	qPCR_R1	GACTAACCCGGAAGTGCAATA				
Primer pair 3	qPCR_F2	CTGCGAGTCCCTTGAAATGTA	62	146	qPCR quantification of *P. cajani*	Consistence results in qPCR
	qPCR_R2	ATACCGCGAATCGAACACTC				
Primer pair 4	qPCR_F3	GGGACGAAAGTCTCTGCTTT	62	110	qPCR quantification of *P. cajani*	Low reproducible results in qPCR
	qPCR_R3	CCTGCAATTCGCATTACGTATC				

#### The standard curve

**Figure 4:**
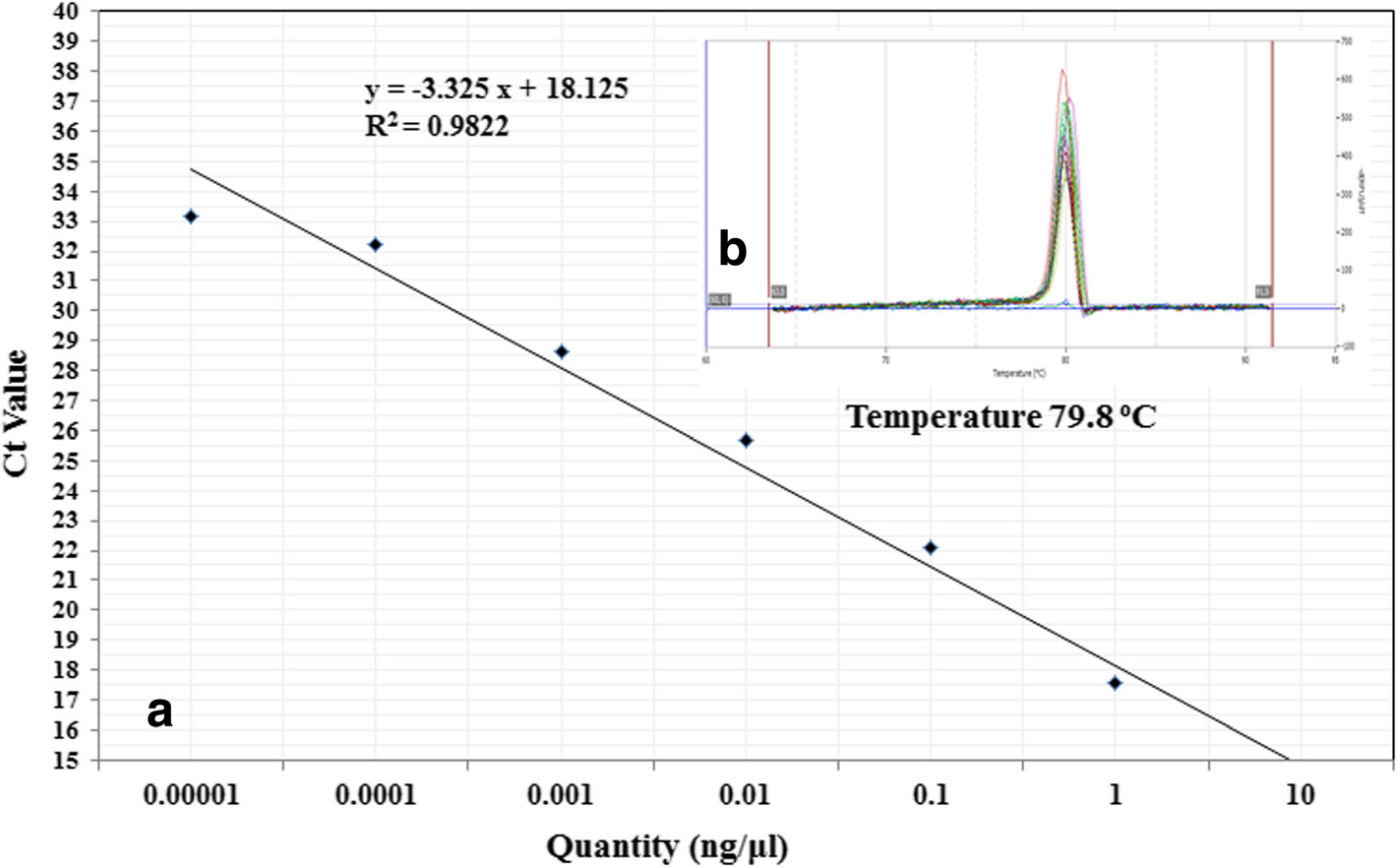
**Calibration of qPCR for quantification of** ***P. cajani*** **: (a)** Standard curve analysis: Standard curve showing the correlation between the log10 DNA amounts (ng) vs. the Ct values for 10 fold dilution of *P. cajani* pure genomic DNA. **(b)** Melting curve analysis: The melting curve (SYBR Green florescence versus temperature) of specific amplifications from the ITS sequences of 5.8S rDNA at different concentrations. The single pick of targeted amplicon at melting temperature (Tm) 79.8°C indicates the specificity of the qPCR primers to *P. cajani*. No contaminating product was detected in PCR reaction.

A standard curve was generated by plotting the cycle threshold value (Ct) versus the logarithm of the concentration of each serial dilution of DNA in a 10 fold over a 7-log range from 10 to 1 × 10^−4^ ng/μL. A good correlation was observed between Ct values and DNA concentration of standard. The slope of linear regression curve was −3.325 with the correlation coefficient R^2^ = 0.9822 demonstrating the PCR efficiency of 99.87% (Figure 4a) Therefore, the standard curve obtained in this study indicated that the nominated primer was highly specific over a linear series of magnitude.

The dissociation analysis showed that the SYBR® Green consistently generated a single peak at 79.8°C in the PCR reactions, demonstrating the presence of only one specific product in the reaction (Figure 4b). The developed qPCR system was used to analyse the amount of *P. cajani* DNA present in an unknown sample by interpolating its Ct value against the standard curve. The qPCR sensitivity result showed at least 0.001 pg fungal DNA was needed for a positive signal in reaction for SYBR-Green detection (results not shown).

#### *Phytophthora cajani* colonization under elevated CO_2_

**Figure 5:**
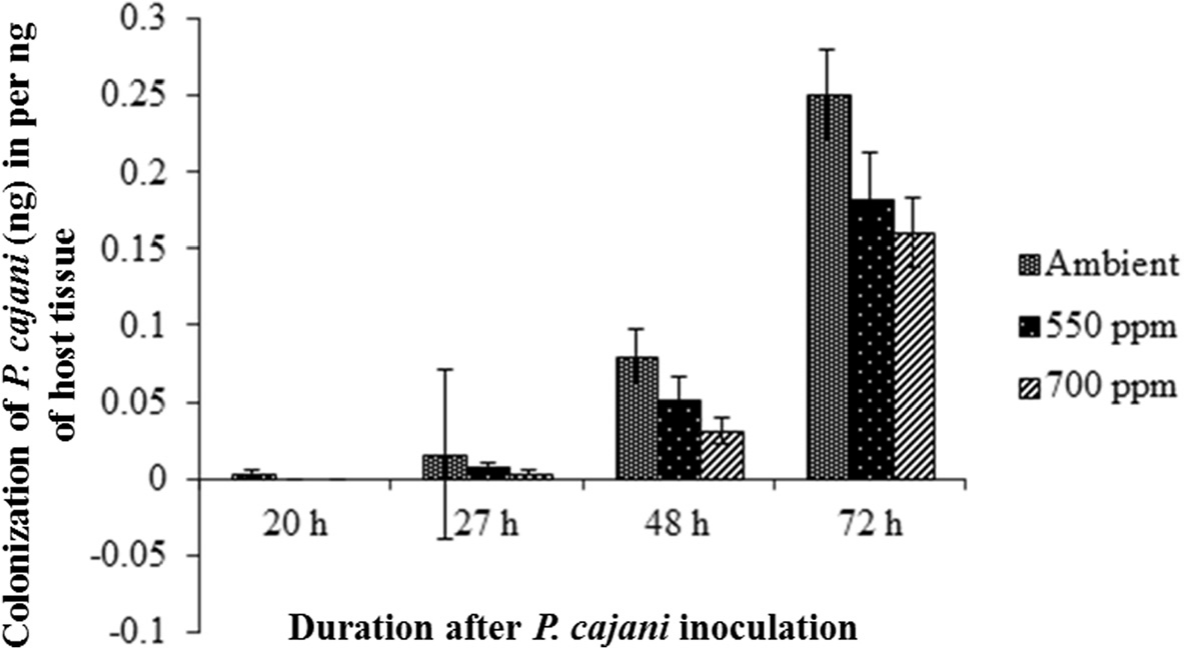
**Chronological colonization profile of** ***P. cajani*** **in root tissues of inoculated pigeonpea (cv. ICP 7119) seedlings grown in three different CO**_**2**_ **conditions, ambient, 550 ppm and 700 ppm respectively.** Absolute quantification of fungal DNA was determined in real time PCR assay using ITS sequences of 5.8S rDNA. Error bar represents the standard error of three biological replicationsat the 95% confidence interval.

Fungal colonization was delayed with the increase of CO_2_ concentration. In ambient condition, the fungal DNA reached to the minimum detection limit 0.001 pg in 1.0 ng of host plant DNA after 20 h of the inoculation with zoospores. In case of elevated CO_2_ concentration of 550 ppm and 700 ppm, the fungal colonization was detected at 27 h post inoculation. The amount of fungal DNA was found to be increased in the host tissues with the time period. The maximum fungal colonization was recorded at 72 h of post inoculation. In ambient, the maximum 0.25 ng of *P. cajani* DNA was detected within the 1.0 ng root DNA of pigeonpea, whereas 0.18 ng and 0.16 ng fungal DNA was measured within the 1.0 ng root DNA of the plants grown in 550 ppm and 750 ppm CO_2_, respectively (Figure 5). No cross reactivity was found with non-inoculated plant samples.

## Discussion

We studied the effect of elevated CO_2_ on PB of pigeonpea. The study included isolation and molecular characterisation of *Phytophthora cajani* and simultaneously developing a standard protocol for zoospore production and reliable inoculation method for *in planta* infection process. Our current study documented first time the molecular identification of *P. cajani* on pigeonpea and further quantified the *P. cajani* in pigeonpea seedlings grown under ambient and elevated CO_2_.

The ITS sequence of rDNA is the most proposed DNA region in molecular fungal ecology and has been recommended as the sole universal fungal barcode for fungi [26]. For *Phytophthora* spp., ITS have been shown to be useful for species identification, although some related species share identical ITS sequences [5,27]. In addition, universal ITS1 and ITS4 primers that has been widely used in most of the labs, was successfully used in this study to identify the *P. cajani* from the isolated fungal samples. In the current study, fourteen *P. cajani* isolates were identified and characterised at molecular level using ITS1 and ITS4 primers from different locations of India. Although *P. cajani* is a novel species, phylogenetic relationship of *P. cajani* with the other taxa of *Phytophthora* based on nucleotide sequence is now well recognized. In our study, it was found that all 14 *P. cajani* isolates grouped into same clade with nearest relative *P. sojae*. Based on DNA distance analysis of the combined ITS1, 5.8S subunit, and ITS2 regions of the genomic ribosomal RNA tandem gene repeat, Cooke et al. [28] showed that *P. cajani* placed into Clade 7b where the nearest relative *P. sojae* does belong. There was no discordance phylogenetic relationship noted within the isolates.

*Phytophthora* species are notorious oomycete pathogen that causes diseases through wide range of inoculum, e.g. mycelium, sporangia, oospore and zoospore. A suitable protocol is required in order to establish a successful infection *in planta*. Several researchers have worked on different *Phytophthora* species to enable an efficacious and repeatable method for *in planta* infection, but for *Phytophthora* sp. on pigeonpea, none of the methods tested in the past have been found consistently reliable (unpublished data). Various methods reported for screening *Phytophthora* in different crops included; *in vitro* leaf disk infection of brussels sprout plants (*Brassica oleracea* var. *gemmifera*) by infectious zoospores of *P. brassicae* [14]; root dip of pea in zoospore suspension of *P. pisi* [29]; leaf detached method in *Nicotiana tabacum* for *P. parasitica* var. *nicotianae* [11]; germination of soybean seeds in soil supplemented with zoospores suspension of *P. megasperma* var. *sojae* [30] and rapid radicle assay in pepper (*Capsicum annuum* L.) for *P. capsici* [31] etc. But no effective screening technique was reported for resistant selection of pigeonpea to *P. cajani*. This is the first time we have established a successful method for obtaining high concentrations of zoospores followed by its use for standardization of inoculation technique in pigeonpea. Use of zoospores to establish *in planta* infection is convenient and suitable to carry out small and large scale inoculation experiments. Testing of previously published techniques [11,14,29-31] with various alterations was carried out, including those found useful for other *Phytophthora* species to develop a suitable method for obtaining high concentrations of *P. cajani* zoospores. Finally, we were successful in developing a repeatable, reliable and economical screening technique using zoospore suspension for PB development.

Visual estimation of infection in the field is time-consuming. Furthermore, clear differentiation of the *Phytophthora* species based on morphology requires expert knowledge. However, with the advent of real-time PCR, plant pathologist possesses the unprecedented ability to accurately quantify the specific pathogen within a host plant [32]. Since last one decade efforts have been made in detection and quantification of various plant pathogens e.g. *Macrophomina phaseolina* [33], Phytoplasma [34], different *Fusarium* species [35], *Phytophthora* species [29]. In this study, real-time PCR assay based on SYBR green chemistry was developed for *P. cajani* quantification.

To determine the effect of elevated CO_2_ on colonization and disease establishment by *P. cajani* in pigeonpea, we targeted 5.8S rDNA to quantify the fungal DNA in host tissue. The sensitivity of the assay was determined using dilution series of pure DNA of known concentration from the *P. cajani*. In general, the detection limit was 0.001 pg per 1.0 ng of host DNA. The reproducibility of the real time assay was evaluated for initial DNA content, presence of PCR inhibitors etc. The low variation between assays and single pick in melting curve indicated high reproducibility of the real-time PCR. However, the variation was eliminated by normalizing samples with the control.

We observed delayed colonization of *P. cajani* and disease establishment under elevated CO_2_. However, pigeonpea seedlings showed no significant difference in disease incidence under elevated and ambient CO_2_ levels. The amount of fungal DNA was found to be increased in the host tissues with the time period and was more in ambient as compared to elevated CO_2_. However, it is not clear that reduction of fungal growth performance is because of negative effects of elevated CO_2_ on fungal performance. Tomato plants showed increased tolerance to infection by *Phytophthora parasitica* at elevated 700 μMmol^−1^ of CO_2_. The results suggested that more vegetative growth in aerial parts and as well as in root systems under higher CO_2_ might have reimbursed the loss of growth caused by the root pathogen [36]. Reduction of powdery mildew (*E. graminis*) on wheat and barley and the severity of rust (*Puccinia sparganioides* Ellis & Barth) are also reported [24]. On the other side, higher threats of *Phytophthora infestans* in potato and diseases of rice, like blast (*Pyricularia oryzae*) and sheath blight (*Rhizoctonia solani*) is increased day to day due to elevated CO_2_ concentration and higher temperature [37]. For instances, it was found that *in vitro* exponential growth rates of *Phyllosticta minima*, a maple fungal pathogen were enhanced by 17% under elevated CO_2_, discounting the possibility that disease reductions by elevated CO_2_ [38].

Increased CO_2_ levels can influence both the host and the pathogen in various means. Under elevated CO_2_, sometimes internal physiological adjustment occurs in plant systems that alter sugar concentration, C:N ratio by producing phenolics compounds in the cells and nutritive quality may lead to unfavourable condition for growth of pathogenic organism and reduced the disease severity. In few study, it was found that elevated CO_2_ increased the thickening of the roots in plants by more production of root border cells [39] and lead to the increased amounts of root exudates into the rhizodeposition [40,41]. The fungal penetration also inhibit in root cells due to thickening of cell walls by deposition in root due to elevated CO_2_. These changes make the environment more favourable for plant defence. As elevated CO_2_ directly involve in change of the vegetative growth of the plants, it is assumed that the amendment of the auxin, cytokinin ratio in plant cells also occurs simultaneously. It is reported that the auxin content modulates the infectivity of *Phytophthora parasitica* in Arabidopsis [42]. Therefore, our further study is aimed to investigate the effect of elevated CO_2_ coupled with other climate variables such as temperature on PB of pigeonpea and investigate the expression of resistance at biochemical level.

## Conclusions

Little is known concerning the isolation method and the infection by zoospores of *Phytophthora cajani*, being a novel species on pigeonpea. The study included isolation and molecular characterisation of *P. cajani* and simultaneously established a standard protocol for zoospore production and *in planta* inoculation technique via isolated zoospores. The zoospore inoculation will facilitate the epidemiological studies and thus will be useful for developing a rapid and reliable screening technique against Phytophthora blight of pigeonpea. qPCR had allowed detection and quantification of *P. cajani* in samples with low pathogen densities. No significant effect of elevated CO_2_ on PB of pigeonpea was observed. To the best of our knowledge, this is the first report of molecular identification of *P. cajani*, zoospore inoculation method and real-time PCR application used to assess the extent of colonization by *P. cajani* during disease development in pigeonpea.

## Methods

### Plant material

The seeds of Pigeonpea cv. ICP 7119 (commonly known as HY-3C) used for this study were procured from the breeding unit of the International Crop Research Institute for the Semi-Arid Tropics (ICRISAT), Patancheru, Telangana State, India. The variety is reported highly susceptible to Phytophthora blight [43].

### Fungal isolates and disease incidence

During a disease survey 2012–13 and 2013–14 *Kharif* seasons, pigeonpea plants exhibiting symptoms of PB were sampled from epidemic block in the field from different locations in the districts of Telangana State and Uttar Pradesh in India (Table 1). Symptomatic plant materials were placed in labelled plastic bags, which were transported in cooler box and stored in 4°C until fungal culture were purified from the samples in the laboratory (Legumes Pathology, ICRISAT). Stem tissues with typical PB lesions were selected for direct isolation of the *Phytophthora*. Symptomatic tissues were cut and surface sterilised by sodium hypochlorite (2% v/v) for 2–3 min followed by washing in sterile distilled water. The tissues were then placed onto V8 juice agar media (Himedia, Mumbai, India) amended with PARP antibiotics (pimarcin 400 μL; ampicillin 250 mg; rifampicin 1000 μL; and pentachloronitrobenzine 5 mlL^−1^ media). Plates were incubated at 25°C in the 12 h/12 h day-night photoperiod for 5 days. Putative *Phytophthora* colonies were transferred to 20% tomato extract agar slants and maintained under *in vitro* at 15 ± 1°C in dark condition by regular sub culturing after 15–20 days. The disease incidence of the individual fields from where the plants sampled were recorded and their details were provided in Table 1. Disease incidence (DI) was calculated by following formula:

$$ \mathrm{D}\mathrm{I}\left(\%\right)=\mathrm{Total}\ \mathrm{number}\ \mathrm{of}\ \mathrm{infected}\ \mathrm{plants}/\mathrm{total}\ \mathrm{number}\ \mathrm{of}\ \mathrm{plants}\ \mathrm{observed}\times 100. $$

The *P. cajani* isolates used in the present study are available with ICRISAT for further use by any scientific community. These isolates are provided based on standard material transfer agreement.

### Morphological identification

Individual isolates were grown on V8 juice agar plate at 25-28°C under continuous fluorescent light. After 5 to 7 days, 5 mm disc of an actively grown hyphal culture was inoculated on 5% tomato juice extract in Petri plates and incubated at 25°C. After 24 hr, the extract was replaced with sterile distilled water and incubated for another 24 hr in the same conditions. After this, the colony and sporangial morphology was observed under the microscope. Other structures, oogonia, antheridia and oospores were also observed on V8 juice agar and tomato agar 2–3 weeks after incubation at 30°C.

### Identification with ITS sequencing

The internal transcribed spacer region (ITS1 and ITS2) including 5.8 s gene of the ribosomal DNA (rDNA) of each isolate was sequenced for DNA-based identification. An agar disk about 5 mm of each isolate grown in V8 juice agar were transferred into 50 mL of tomato juice broth in a 250 mL flask for 5 to 7 days at 25°C. Mycelial mats were harvested by discarding the media and dried on autoclaved blotting paper. Dried mycelial mat was stored at −80°C. Genomic DNA (gDNA) was extracted from mycelia of *Phytophthora* with the PureLink Plant Total DNA Purification kit (Invitrogen, USA) as per manufacturer’s protocol using 100 mg of fungal tissue. The purified DNA was evaluated in 0.8% (w/v) agarose gel stained with 0.2 μg/mL ethidium bromide and visualized under UV light. The quantity and quality of total DNA was determined by the OD_260/280_ using Nanodrop spectrophotometer (Thermo Scientific, USA). The extracted DNA was stored at −20°C.

PCR amplification of the ITS regions (ITS1 and ITS2) and 5.8S ribosomal DNA was performed with the ITS1 (F) and ITS4 (R) (Table 2) [44]. Reactions were performed in 50 μL volumes composed of 5 μL of 10X PCR buffer, 3.0 μL of 50 mM MgCl_2_, 1.0 μL of 10 mM dNTP mix (2.5 mM of each dNTP), 0.5 μL of each 10 mM primer, 0.5 μL of 5 U/μLTaq DNA polymerase Brazilian origin (Invitrogen, USA), 1 μL of extracted DNA and nuclease-free water for volume make up to 50 μL. Thermal cycling conditions consisted of an initial denaturation of 94°C for 5 min; 35 cycles of 94°C for 30 s, 55°C for 30 s, and 72°C for 1 min; and a final extension step of 72°C for 10 min. Amplified products were confirmed and concentrations estimated visually in with 1% agarose gel electrophoresis. The PCR products were then sequenced in an automated sequencer using ITS1(F) and ITS4 (R) by a commercial service.

### Phylogenetic analysis

The DNA sequences of all *P. cajani* isolates were aligned in BioEdit v. 7.2.5 and edited manually for generating consensus sequences. The sequence of each isolate was subjected to BLAST analysis using the database of *Phytophthora* (www.phytophthoradb.org) and GenBank (NCBI, Bethesda, MD). The sequences were then submitted to NCBI data base. Multiple sequence alignment was performed with CLUSTAL X version 1.81 and pairwise sequence identities among the *P. cajani* isolates were compared using Gene Doc version 2.6.002. Phylogenetic tree was constructed with 35 other *Phytophthora* spp. as out groups using the program MEGA6.06. Model was predicted by JModeltest 2.1.7 v20141120 software. The best suited model for the data was NrT + G + I on the basis of BIC, AIC and DT scores [45]. The tree was constructed with Maximum Likelihood statistical mode using the NT93 with gamma (G) plus invariable parameter (I) and taking 1000 replications for bootstrap value.

### Standardization of zoospore production

For *in planta* infection of pigeonpea seedlings, method for preparation of zoospores suspension culture from *P. cajani* was standardised. Isolate ICPC 1 was chosen as it was consistently virulent on susceptible genotype. One piece (5 mm) of mycelial mat from pure culture of isolate ICPC 1 was inoculated in 100 ml conical flasks containing 25 mL 5% tomato extract broth and incubated in darkness at 25°C for 72 h. After post inoculation period, the tomato extract broth was decanted and replaced with 25 mL sterile pond water, which was immediately decanted again and replaced with 25 mL fresh pond water, in which the mycelium was further incubated at 25°C in dark for 4 h. The procedure was repeated soon after the completion of incubation period and incubated for 20 h, in these cases with single changes of water. Finally, mycelial growth of isolate was removed from the flask and zoospores were harvested in water suspension. The zoospores were checked under microscope and the concentration of zoospores was measured in a haemocytometer. The suspension was used for *in planta* infection with further dilution.

### *In planta* infection system under elevated CO_2_

The apparently healthy seeds of ICP 7119 were surface sterilized in sodium hypochlorite (1% v/v) for 2–3 min and then washed in sterile double distilled water. The seeds were sown (1 seed/well) in seed trays filled with autoclaved sand:vermiculite (9:1) mixture at 2 cm depth and well watered. The trays were then divided into three different sets, each treatment with three replications with 50 seedlings per tray and kept in three separate plant growth chambers adjusted with three different experimental conditions. Plant growth chambers were tuned to adjust different CO_2_ concentration and termed as E1 for ambient (~380 ppm), E2 for elevated CO_2_ to 550 ppm and E3 for elevated CO_2_ to 700 ppm, and maintained at 28°C with >75% humidity. After seven days, seedlings were inoculated with *P. cajani* zoospore suspension (1.5 × 10^5^ zoospores/mL) so that the roots were submerged up to collar region in each well. Similar number of seedlings inoculated with only sterilized water served as un-inoculated control. The experiment was designed in completely randomized design (CRD) manner and disease incidence was recorded everyday up to the complete mortality. The critical differences (CD) value of disease incidence was calculated at 1% level at 36 h, 48 h and 72 h.

For quantification of *P. cajani* infection *in planta*, seedlings were harvested 0 h (immediate after infection), 2 h, 20 h, 27 h (30), 48 h and 72 h of post inoculation, washed and preserved in −80°C for further experiments.

### Total genomic DNA (gDNA) extraction from plant samples

Total gDNA from *Phytophthora* infected plant samples was isolated using PureLink Plant Total DNA Purification kit (Invitrogen, USA) as per manufacturer’s protocol. About 100 mg of root tissue was ground in liquid N_2_ and resuspended in 250 μL Resuspension Buffer (supplied in the kit). The tissues were homogenized with vigorous vortexing until sample was completely resuspended. About 15 μL 20% SDS and 15 μL RNase A (20 mg/mL) were added to the tissue resuspension and incubated at 55°C for 15 minutes to complete lysis of tissues. Total gDNA was eluted in 50 μL of Elution Buffer and stored at −20°C for further downstream application. The purified DNA was evaluated in 0.8% agarose gel as well as by UV spectrophotometry. The extracted DNA was stored at −20°C.

### Development and evaluation of qPCR primers

To quantify the growth of *P. cajani* within the host tissue under different experimental conditions (E1, E2 and E3), the real-time PCR was carried out. Three pairs of sequence specific primers were designed from internal transcribed spacer (ITS) sequences of *P. cajani* using IDT Primer Quest software (eu.idtdna.com/Primerquest/Home/Index). ITS sequences (Acc nos. KJ010534-KJ010538) were aligned using BioEdit v. 7.2.5. and primers were designed from conserved region of the ITS sequences. To test the specificity of the primers pairs, PCR was carried out separately with the individual primers sets using pure gDNA of *P. cajani* in 50 μL reaction mixture containing: 5 μL of 10 X *Taq* polymerase buffer, 1.5 μL 50 mM MgCl_2_, 1 μL of 10 mM each dNTP, 1 μM of each primer, 1 μL of 5u/1 μL *Taq* polymerase (Invitrogen, USA), about 100 ng of gDNA and H_2_O up to 50 μL. Reaction conditions were: 94°C for 4 min, (94°C for 45 s, 60°C for 45 s and 72°C for 30 s) × 35 cycles followed by incubation at 72°C for 10 min. The amplified products were separated by 1.5% (w/v) gel electrophoresis and sequenced. The expected size of PCR amplicons was constantly generated from the real-time PCR analysis.

### Real-time PCR reaction

Real-time PCR was carried out in a total volume of 20 μL consisting of 10 μL 2x SYBR Green PCR Master Mix (Applied Biosciences, USA), 500 nM of each primer (qPCR_F2 and qPCR_R2) (Table 2), and 1 ng of each template DNA. Sterile bi-distilled water was added up to a final volume of 20 μL. The PCR thermal cycling conditions were as follows: 50°C for 2 min, 95°C for 10 min, 40 cycles of 95°C for 10 s, and 62°C for 30 s (during which the fluorescence was measured). Following the final amplification cycle, a melting curve was constructed by measuring the fluorescence continuously when heating from 60 to 95°C at the rate of 0.5°C per second. qPCR reactions were performed in a PikoReal 24 Real-Time PCR Detection System (Thermo Scientific, USA). To generate the standard curve, DNA from pure culture of *P. cajani* was subjected to qPCR with a 10-fold dilutions ranging from 10 ng to 0.01 pg under the same conditions described above. Quantification values were automatically determined by the PikoReal software version 2.0 (Thermo Scientific, USA) and the threshold cycle (Ct) values were then obtained. The standard curve is a plot of the Ct versus log DNA concentration. In all the experiments, appropriate negative controls containing no template were subjected to the same procedure to eliminate or to detect whether any possible DNA contamination present. Each sample was amplified in triplicate replication in every experiment.

## Abbreviation

ITS: Internal transcribed spacer
qPCR: Quantitative polymerase chain reaction
PB: Phytophthora blight
rDNA: Ribosomal DNA
ICRISAT: International Crops Research Institute for the Semi-Arid Tropics
PARP: Pimarcin ampicillin, rifampicin and pentachloronitrobenzine
DI: Disease incidence
gDNA: Genomic DNA
UV: Ultra violet
BLAST: Basic local alignment search tool
NCBI: National Center for Biotechnology Information
MEGA6.06: Molecular evolutionary genetics analysis version. 6.06
CRD: Completely randomized design
CD: Critical differences
IDT: Integrated DNA technologies
Ct: Cycle threshold
